# Agent-based modeling identifies multiple paths for inflammation resolution

**DOI:** 10.1093/bioinformatics/btag533

**Published:** 2026-08-19

**Authors:** Hermès Desgrez-Dautet, David Bernard, Béatrice Cousin, Paul Monsarrat, Sylvain Cussat-Blanc

**Affiliations:** CNRS UMR 5070, INSERM UMR 1301, Institut RESTORE, Toulouse 31100, France; Université de Toulouse, Toulouse 31400, France; Institut de Recherche en Informatique de Toulouse (IRIT), Toulouse 31400, France; Institut de Recherche en Informatique de Toulouse (IRIT), Toulouse 31400, France; Université Toulouse Capitole, Toulouse 31000, France; CNRS UMR 5070, INSERM UMR 1301, Institut RESTORE, Toulouse 31100, France; Université de Toulouse, Toulouse 31400, France; CNRS UMR 5070, INSERM UMR 1301, Institut RESTORE, Toulouse 31100, France; Université de Toulouse, Toulouse 31400, France; Dental Faculty, Toulouse, France; Institut de Recherche en Informatique de Toulouse (IRIT), Toulouse 31400, France; Université Toulouse Capitole, Toulouse 31000, France; Institut Universitaire de France, France

## Abstract

**Summary:**

Sterile inflammation emerges from spatially coordinated interactions between immune recruitment, polarization, signal diffusion, and tissue turnover. Capturing this complexity requires modeling frameworks that integrate stochasticity, spatial heterogeneity, and functional redundancy across immune cell types. We present a spatially explicit agent-based model of sterile inflammation based on a functional mono-agent abstraction, in which immune cells share core capabilities, e.g. polarization, chemotaxis, signal production, and phagocytosis, while differing through parameterization rather than rigid rule sets. Using large-scale global parameter exploration via Latin Hypercube Sampling, we generated 300 000 simulations to characterize the model’s dynamical landscape. Dimensionality reduction suggested a continuous manifold of inflammatory trajectories. Applying biologically informed temporal criteria identified a highly constrained subspace consistent with canonical sterile inflammation. Within this region, multiple distinct yet self-consistent, kinetic regimes achieved successful resolution. These results indicate that sterile inflammation is not a single dynamical attractor but an emergent coordination constraint in a high-dimensional functional space, providing a systems-level framework for exploring immune regulation and dysregulation.

**Availability and implementation:**

Model can be found as a demo on ISiCell website at https://isicell.irit.fr/app.html.

## 1 Introduction

Population aging represents a major public health challenge. One of the central hallmarks of the aging process is the emergence of a persistent low-grade inflammatory state, commonly referred to as inflammaging. This chronic inflammatory background is associated with impaired tissue repair, immune dysregulation (Li *et al.* 2023), and progressive functional decline across organs (Kemoun *et al.* 2022, López-Otín *et al.* 2023, Karpuzoglu *et al.* 2025). Inflammaging does not arise from a single molecular pathway (Hu *et al.* 2023) but from complex interactions among immune cells, stromal components, damaged tissue, and diffusible molecular signals (Müller and Di Benedetto 2025, Spray *et al.* 2025). Although inflammatory responses are tissue-specific in molecular composition, their functional mechanisms—activation, polarization, recruitment, clearance of dead cells, and resolution—are largely conserved. Understanding how these interacting mechanisms regulate inflammation resolution, and how their alteration induces chronic inflammation remains an open systems-level question.

Experimental approaches, particularly longitudinal in vivo studies, have provided essential insights into inflammatory processes (Těšický *et al.* 2021, Mastrogiovanni *et al.* 2024). However, they remain costly and limited in their capacity to explore the large combinatorial space of mechanistic hypotheses. Computational modeling offers a complementary framework to formalize biological assumptions and investigate the dynamical consequences of interacting mechanisms. Differential equation–based models provide quantitative descriptions of well-characterized interactions in a deterministic schema (Alt and Lauffenburger 1987, Alakesh *et al.* 2022), while data-driven approaches extract predictive patterns from large-scale datasets (Kalyakulina *et al.* 2025, Sim *et al.* 2025). Yet both paradigms face limitations when spatial heterogeneity, stochasticity, and incomplete mechanistic knowledge are central to the system. Inflammation, in particular, depends strongly on local cell–cell and cell–environment interactions. Agent-Based Modeling (ABM) provides an alternative framework by representing individual cells as interacting agents within a spatial environment. ABMs naturally capture stochasticity, spatial structure, and emergent collective behavior while allowing explicit encoding of mechanistic rules at the cell and environment level.

In classical inflammatory ABMs, macrophages, monocytes, neutrophils, and lymphocytes are most commonly represented, alongside additional actors depending on the specific mechanisms studied. These immune cell types are typically represented as distinct entities with specific rule sets (Segovia-Juarez *et al.* 2004, Folcik *et al.* 2011, Gopalakrishnan *et al.* 2013, Seekhao *et al.* 2018, Nickaeen *et al.* 2019, Uleman *et al.* 2023). In this work, we focus on resident and circulating macrophages (including monocytes), neutrophils, and mesenchymal stromal cells (MSCs), included for their well-documented immunoregulatory roles. Additionally, many immune populations like these share overlapping functional capabilities, including polarization, cytokine secretion, chemotaxis, and phagocytosis (Ma *et al.* 2016, Planat-Benard *et al.* 2021, Strizova *et al.* 2023). This functional redundancy raises the question of whether inflammatory dynamics can be understood at a higher level of abstraction, focusing on shared functional profiles rather than strictly defined cellular identities [e.g. Sharpee works on neurons (Sharpee 2014)].

Here we present an agent-based model of sterile inflammation, grounded in a mono-agent conception, that is to say a multi-agent model with shared functional behavior. This novel framework captures the diversity of cellular profiles while preserving the intrinsic plasticity and flexibility of cell states. Our architecture is defined by four core capabilities—polarization, chemotaxis, signal production, and phagocytosis—each implemented by every simulated agent. Biological diversity among immune cell types emerges not from distinct behavioral frameworks, but from specific parameter regimes within this common functional space. We combine this framework with large-scale global parameter exploration to characterize the dynamical landscape of inflammatory responses and identify constrained regimes compatible with canonical sterile inflammation. Such results validate the model’s capacity to provide a suitable representation of inflammation.

## 2 Materials and methods

### 2.1 Modeling platform

To develop our agent-based model, we used the ISiCell platform, a modeling environment that enables graphical design of multi-agent systems (Cogoni *et al.* 2024). This approach facilitates interactions with biologists by making local rules, cellular interactions, and spatial organization explicit. ISiCell supports simulations in arbitrary dimensions. In this study, we modeled a two-dimensional tissue slice, allowing multiple agents to occupy the same grid cell, providing a compromise between spatial realism and computational efficiency. Agent-produced signals are represented on a dedicated grid with the same resolution as the simulation lattice. These signals diffuse locally to neighboring cells, enabling spatial propagation of inflammatory information.

### 2.2 Mono-Agent model of inflammation

#### 2.2.1 Environment

**Figure 1:**
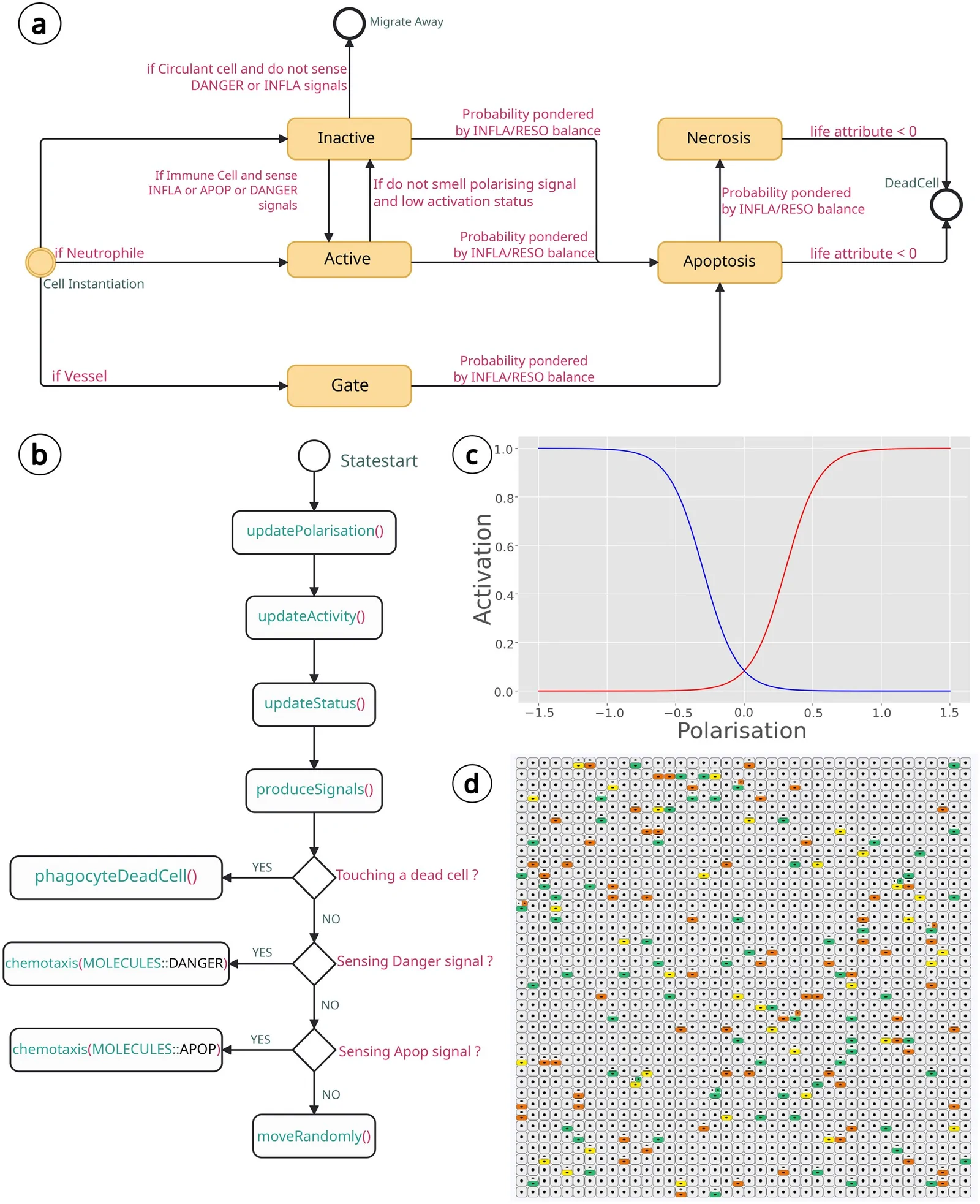
Construction of the model. The model was developed using ISiCell, a dedicated platform that provides a graphical representation of the underlying code used to implement agent rules. (a) State-transition diagram of the model. Cells are instantiated in the *Cell Instantiation* state. The *Migrate Away* and *Deadcell* states correspond to cell removal. (b) Activity diagram of the *Active* state. Functions are executed sequentially from *updatePolarisation* to *produceSignal*; the final action depends on priority rules that match the current environment. (c) Sigmoid system used to represent polarization and activity levels. The blue curve translates polarization into resolutive activity, whereas the red curve corresponds to inflammatory activity. Both the slope and the offset are parameterized. (d) Example of the grid at step 1 after initialization. Grey cells correspond to *Others*, yellow to *Vessels*, orange to *Resident Macrophages*, and green to *Stromals*. *Neutrophils* (purple) and *Circulating Macrophages* (red) are absent at initialization.

The simulated tissue is represented as a two-dimensional 40 × 40 lattice (Fig. 1d). Each grid position corresponds to a spatial location that may host multiple agents simultaneously (e.g. one adipocyte and the surrounding cells). This allows simplified local density variations and contact-based interactions. To obtain a tractable representation of a tissue cross-section, the grid was given a toroidal topology along its width in order to avoid edge effects.

#### 2.2.2 Agent taxonomy

Depending on their initial parameters, agents (cells) are tagged to six types: resident macrophages, circulating macrophages, neutrophils, MSCs, blood vessels, and generic tissue cells (“Other”). The first four are immune-functional agents governed by the mono-agent framework, sharing a common set of functional capabilities while differing through parameterization (Table S1, available as supplementary data at *Bioinformatics* online). Blood vessels act as recruitment gates, whereas generic cells serve structural roles. Vessels do not reproduce realistic vascular geometry but spatially localize recruitment and introduce stochasticity in circulating cell entry.

**Table 1:** Implemented states, associated functions, and conditions to enter the state.

State	Function	Access conditions
Inactive	Move randomly.	No inflammatory, apoptosis, or danger signal perceived. Is circulating macrophage when at recruitment. Is resident macrophage, MSC or “Other” at initialization.
Active	Update polarization index and activity levels. Produce inflammatory and/or resolutive signals. Perform phagocytosis or efferocytosis, or go up a signal gradient, or move randomly.	Inflammatory, apoptosis, or danger signal perceived. Is neutrophil at recruitment.
Apoptosis	Produce apoptosis signal.	Depends on the probability to enter in apoptosis influenced by inflammatory/resolutive balance.
Necrosis	Produce necrosis and inflammatory signal.	Depends on the probability to enter in necrosis influenced by inflammatory/resolutive balance.
Gate	Recruit circulating cells accordingly to the inflammatory/resolutive balance.	Is blood vessel at initialization.

#### 2.2.3 Signal representation and diffusion

Inflammatory communication is modeled through four abstract signal categories:

- *Inflammatory signal*: represents pro-inflammatory mediators produced by activated cells (e.g. IL-1, IL-6, TNF-α, IFN-γ, ROS).
- *Resolving signal*: includes mediators promoting inflammation attenuation and transition toward pro-resolving states (e.g. TGF-β, IL-10, IL-4, Annexin-A1).
- *Danger signal*: mimics damage-associated molecular patterns (DAMPs) released by necrotic cells.
- *Apoptotic signal*: represents signals emitted by apoptotic cells, analogous to “find-me” cues attracting phagocytes.

Signals are stored on a dedicated grid matching the cellular lattice resolution. At each time step, they diffuse locally, undergo partial degradation, and are updated before cellular decision processes. This spatial signal layer enables chemotaxis toward danger or apoptotic gradients and locally modulates activation and polarization.

#### 2.2.4 Agent state-transition scheme

Each agent evolves according to a discrete state-transition architecture comprising five states (Fig. 1a, Fig. S1, available as supplementary data at *Bioinformatics* online): inactive, active, apoptosis, necrosis, and entry. Transitions depend on the local inflammatory-to-resolving signal balance and agent type probabilistic parameters modulated by Table 1. At initialization, blood vessels are assigned to the entry state, while generic tissue cells, resident macrophages, and MSCs are inactive. Circulating immune cells are absent at baseline and appear only through recruitment via vessels. The grid is initially composed predominantly of other tissue cells, with fixed proportions of MSCs (4%), resident macrophages (4%), and vessels (2%) randomly distributed. During simulation, immune-functional agents switch between inactive and active states according to environmental conditions. All cells are subject to probabilistic apoptosis, which may progress to necrosis under inflammatory dominance.

#### 2.2.5 Activation logic

Immune-functional agents transition from the inactive to the active state when they detect at least one activating signal in their local environment, namely inflammatory, danger, or apoptotic signals. Signal detection is spatially local and depends on the concentrations present at the agent’s position. Neutrophils differ slightly in that they are activated immediately upon recruitment and therefore enter the tissue directly in the active state. Conversely, active agents may return to the inactive state when activating signals are no longer detected and when their internal activation level (linked to their inflammatory-resolutive polarization) decreases below a defined threshold. This reversible transition enables dynamic adaptation to changing local inflammatory contexts.

#### 2.2.6 Active state behavior

Agents in the active state execute a sequence of operations at each time step (Fig. 1b, Fig. S1b, available as supplementary data at *Bioinformatics* online). They first update their polarization status based on the current balance of inflammatory and resolving signals in their environment. This polarization influences their activation level and modulates the efficiency of downstream functions. Activated agents then produce signals according to their polarization profile, generating inflammatory and/or resolving signals that contribute to the evolving molecular landscape of the tissue. Following signal production, agents evaluate their local environment to determine their movement and clearance behavior. If they are in contact with a dead cell, they may attempt phagocytosis or efferocytosis. In the absence of direct contact, they assess local signal gradients. Detection of danger signals triggers movement toward the corresponding gradient, while detection of apoptotic signals induces movement toward apoptotic signal sources. If no relevant gradient is perceived, agents perform random migration. At the end of each time step, agents update their status, including the possibility of entering apoptosis according to probabilistic rules influenced by the local inflammatory-to-resolving balance.

#### 2.2.7 Polarization dynamics

Polarization is modeled as a continuous internal variable representing the balance between pro-inflammatory and pro-resolving gradient experience (Fig. 1c). Its evolution is governed by two sigmoidal functions: one corresponding to the pro-inflammatory domain and the other, inversely shaped, corresponding to the pro-resolving domain. Exposure to inflammatory signals shifts the polarization index toward positive values, whereas resolving signals shift it toward negative values. The magnitude of polarization determines the agent’s activation level and modulates the intensity of its functional outputs, including signal production and phagocytic efficiency. This formulation enables gradual and reversible transitions between inflammatory and resolving phenotypes rather than discrete state switching, thereby capturing intermediate activation states.

#### 2.2.8 Cell death and signal generation

At each time step, all cellular agents are subject to a probability of entering apoptosis. This probability is weighted by the local balance between inflammatory and resolving signals (inflammatory dominance increases the likelihood of cell death). Apoptotic cells generate apoptotic signals, which serve as attractant cues for phagocytic cells. Apoptotic cells may subsequently transition to necrosis with a probability influenced by exposure to inflammatory signals. Necrotic cells release both danger and inflammatory signals, thereby contributing to the amplification of local inflammatory activity. Through this mechanism, the inflammatory environment directly modulates tissue turnover and signal production, creating feedback between cell death and immune activation.

#### 2.2.9 Recruitment mechanisms

Blood vessels remain permanently in the entry state and serve as spatially localized recruitment gates for circulating immune cells. Recruitment occurs probabilistically and is modulated by the local balance between inflammatory and resolving signals. A predominance of inflammatory cues increases the likelihood of recruitment events. Neutrophils enter the tissue in the active state upon recruitment, whereas circulating macrophages enter in the inactive state and must subsequently detect activating signals to become active. This distinction introduces differences in the temporal dynamics of the recruited populations and contributes to sequential phases of the inflammatory response.

### 2.3 Parameter exploration

The model includes 134 parameters that required investigation. Parameter sets generated to perform the sensitivity analysis were defined using a Latin Hypercube Sampling (LHS) strategy (Stein 1987) to uniformly sample the multidimensional parameter space.

### 2.4 Standardization

Kinetic data were min/max normalized (scikit-learn). The matrix *X* is organized with simulations as rows and time and agent types as columns. Standardization is performed separately for each type. Normalization was performed as


(1) $$X_{\text{scaled}}=\frac{X-\text{min}(X_{\text{type}})}{\text{max}(X_{\text{type}})-\text{min}(X_{\text{type}})}(b-a)+a,$$

where *a* and *b* denote the bounds of the target interval.

### 2.5 Dimension reduction

Principal Component Analysis (PCA, scikit-learn v1.7.2) was performed on vectorized kinetics. The vectors were built by concatenation of immune cell amount dynamics sampled in order to keep only the counts every 10 steps (1 h) (Fig. S2, available as supplementary data at *Bioinformatics* online). The number of components retained was determined to be the minimum required to explain 95% of the total variance. For the purpose of visualization, only the first two principal components were displayed.

**Figure 2:**
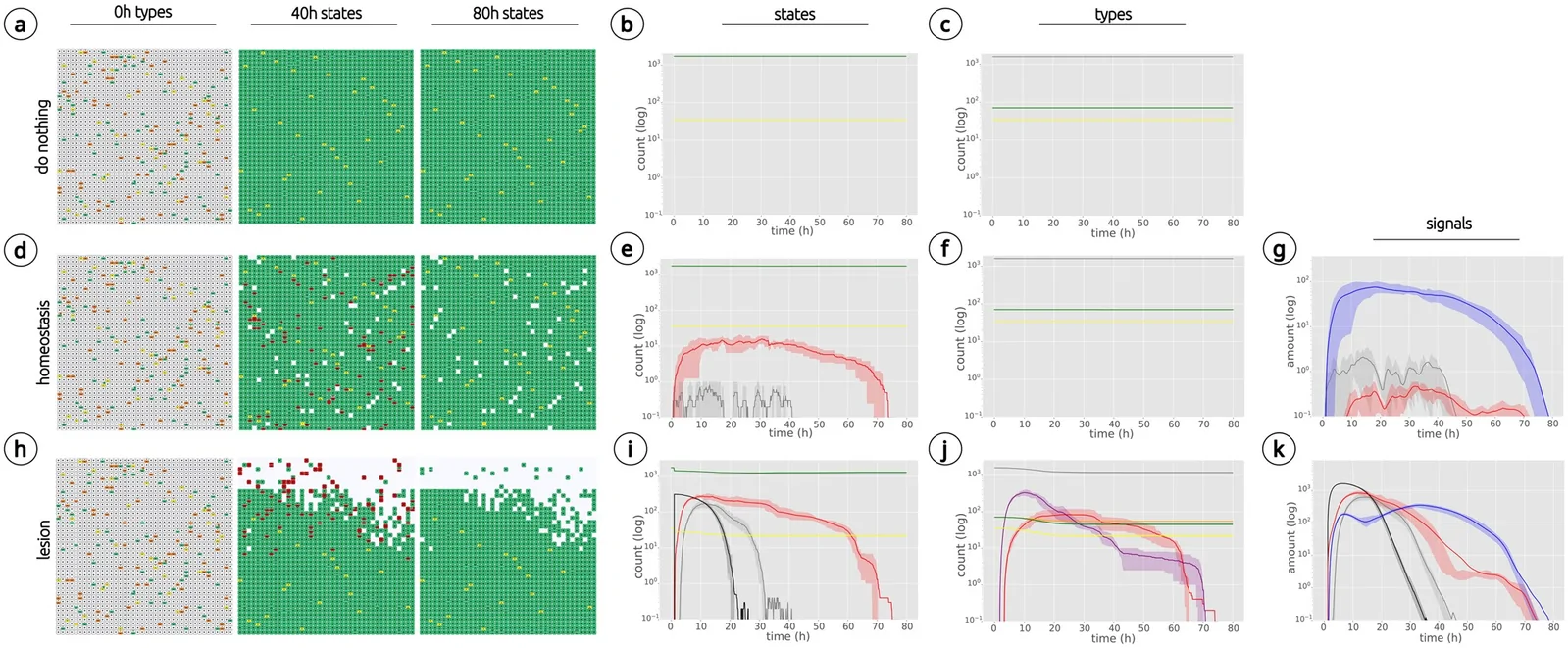
Hand testing the model on various scenarios. Three scenarios are tested, from top to bottom of the figure: no intervention at all, inducing apoptosis randomly first half of the simulation and lesion (necrosis) induced at 1 h. (a, d, h) screenshots of grids at 0 (start), 40 (half) and 80 h (end). The first visualizations are type-colored (see Fig. 1), others are state-colored: green = *Inactive*, yellow = *Gate*, red = *Active*, grey = *Apoptosis*, and black = *Necrosis*. For the next figures, 10 replicates were made, means (solid line) calculated, and percentiles shown (light areas). (b, e, i) are state counts during simulations (same colors as states screenshot). (c, f, j) are type counts during simulation (same colors as types screenshots). (g, k) are total signal amount during simulations, inflammatory = red, resolutive = blue, apoptosis = grey and danger = black.

### 2.6 Clustering

Clustering was performed on the principal components from the PCA, using the clustering methods provided by the scikit-learn (v1.7.2) Library (Fig. S3, available as supplementary data at *Bioinformatics* online). KMeans was preferred as no split structures were observed in the reduced space. The optimal number of clusters was estimated using the Elbow method implemented in Yellowbrick (v1.5), based on the evolution of within-cluster distortion. The cluster stability was evaluated with bootstrapped Jaccard scores.

**Figure 3:**
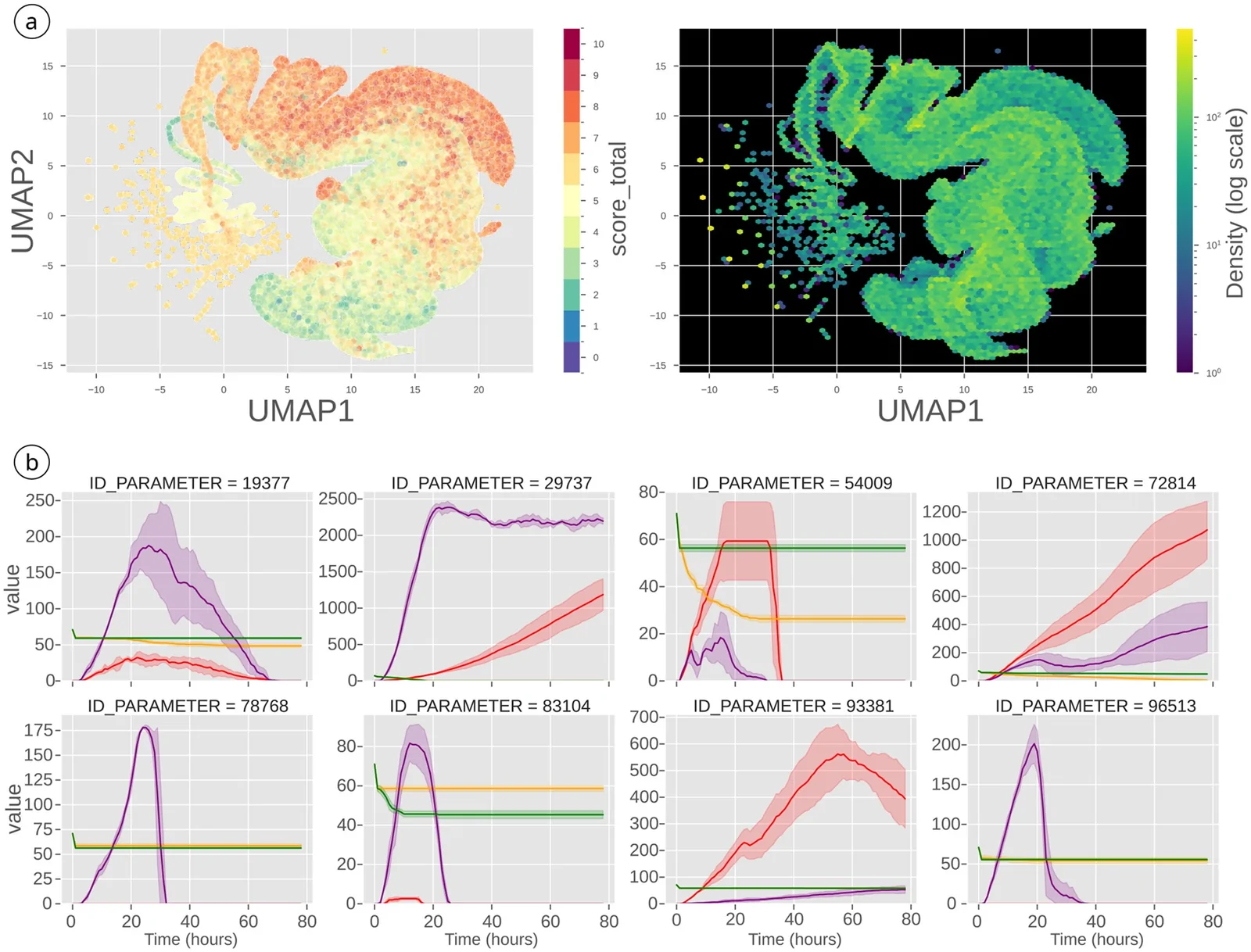
LHS- and expert rule-directed sensitivity analysis. 300 000 LHS simulations were launched (288 575 terminated). Ten tests were applied, the simulation score was defined as the number of tests passed. (a) UMAP of PCA extracted from LHS-derived kinetics of Neutrophils, Macrophages (both) and MSCs, colored by total score (left) and density of points (right), (b) Illustrative samples of all simulations, *Neutrophils* (purple), *Circulating Macrophages* (red), *Resident Macrophages* (orange), and *MSCs* (green).

## 3 Results

### 3.1 Model validation: homeostasis and inflammatory scenarios

To assess whether the model is able to reproduce biologically coherent dynamics (Dwyer and Turnquist 2021), we first evaluated its behavior under controlled scenarios designed to mimic homeostasis and inflammation (Tables S2 and S3, available as supplementary data at *Bioinformatics* online).

#### 3.1.1 Baseline homeostasis

In the absence of any external perturbation, the system remains in a stable homeostatic state. No inflammatory, apoptotic, or danger signals are present in the environment (Fig. 2a–c). Under these conditions, immune-functional agents remain inactive, no recruitment occurs through blood vessels, and signal concentrations remain negligible throughout the simulation. This confirms that the model does not spontaneously generate inflammatory activity in a neutral context and that activation requires explicit triggering signals.

#### 3.1.2 Homeostatic apoptotic turnover

To reproduce physiological tissue turnover, we implemented a scenario in which cells randomly enter apoptosis during the first half of the simulation (Fig. 2d–g). Apoptotic cells generate localized apoptotic signals that activate nearby sentinel cells. After activation, these cells exhibit weak polarization and produce low levels of inflammatory and resolving mediators. Following efferocytosis, their polarization progressively shifts toward a pro-resolving profile, increasing resolving signal production and promoting similar polarization in neighboring activated agents. As apoptotic cells are cleared and no new apoptotic events occur, signal levels decrease. Immune cells gradually depolarize and return to an inactive state, restoring tissue quiescence. This scenario shows that the model produces transient activation followed by self-limited resolution in response to physiological turnover.

#### 3.1.3 Simulation of acute inflammation

To simulate acute inflammation, we implemented a lesion scenario in which necrosis is induced, after a few time steps, in a horizontal band at the top of the grid (Fig. 2h–k). Necrotic cells release inflammatory and danger signals, triggering activation of resident immune cells and recruitment of circulating populations. In the early phase, recruited cells predominantly adopt a pro-inflammatory profile. During the first 30 h, this phase is marked by active clearance of necrotic and apoptotic cells and elevated inflammatory signals. As the dead-cell burden declines, polarization progressively shifts toward pro-resolving states. Resolving signals become dominant, recruitment decreases, and signal levels return toward baseline. By the end of the simulation, dead cells are cleared and circulating immune cells have largely exited the tissue, indicating successful resolution.

### 3.2 Global exploration reveals a constrained dynamical landscape of inflammation

LHS was used to explore the parameter space. This method provides homogeneous coverage of a high-dimensional space while preserving variability across parameters and capturing nonlinear interactions (Tables S4 and S5, available as supplementary data at *Bioinformatics* online). A total of 100 000 parameter sets were generated, each with three stochastic replicates. After excluding simulations exceeding a predefined population size threshold, 288 575 runs corresponding to 96 431 distinct parameter sets were retained. For each simulation, population time series and aggregated signal dynamics were recorded, yielding a high-dimensional representation of inflammatory trajectories.

#### 3.2.1 Inflammation: a continuous dynamical landscape

Principal Component Analysis (PCA) dimension reduction applied to the full set of simulations revealed a continuous distribution of trajectories without clearly separated regimes (Fig. 3a). With a homogeneous sampling of parameter space, the model generates a broad spectrum of inflammatory behaviors forming a smooth dynamical manifold rather than discrete clusters.

This observation suggests that sterile inflammation, as encoded in the model, does not rely on a small number of intrinsic attractors. Instead, inflammatory dynamics emerge from distributed parameter interactions, producing graded transitions between weak, excessive, delayed, or rapidly resolving responses. In this sense, the model behaves as a high-dimensional dynamical system whose macroscopic outcomes reflect coordinated microscopic rules.

#### 3.2.2 Biologically plausible inflammation as an emergent constrained region

To identify trajectories consistent with canonical sterile inflammation, we defined expert-based and biologically informed criteria capturing key characteristics of the inflammatory response (Supplementary methods, available as supplementary data at *Bioinformatics* online). Neutrophils were required to exhibit an early peak (6–48 h), followed by recruitment of circulating monocytes/macrophages. Inflammatory signals had to progressively decline and be replaced by resolving signals, and resolution required clearance of dead cells together with the disappearance of most recruited circulating cells (Dwyer and Turnquist 2021, Guillamat-Prats 2021).

Among the 288 575 simulations, only 699 met all criteria, corresponding to 413 distinct parameter sets (Fig. S2a, available as supplementary data at *Bioinformatics* online), defining a highly restricted region of the explored space. This restriction did not depend on a single dominant parameter but on specific combinations of interacting parameters (Fig. S2e, available as supplementary data at *Bioinformatics* online). Plausible inflammation kinetics therefore occupies a constrained submanifold within a broader dynamical landscape. Although the model generates diverse inflammatory regimes, canonical resolution emerges only under coordinated regulation of recruitment, polarization, signal production, and clearance (Fig. 3b). Resolution thus appears not as a default outcome but as an emergent property arising from balanced pro-inflammatory and pro-resolving interactions.

### 3.3 Multiple inflammatory regimes within the biologically plausible subspace

Having shown that biologically plausible dynamics occupy a restricted region of the global landscape, we next examined whether this region was homogeneous or structured into distinct regimes.

**Figure 4:**
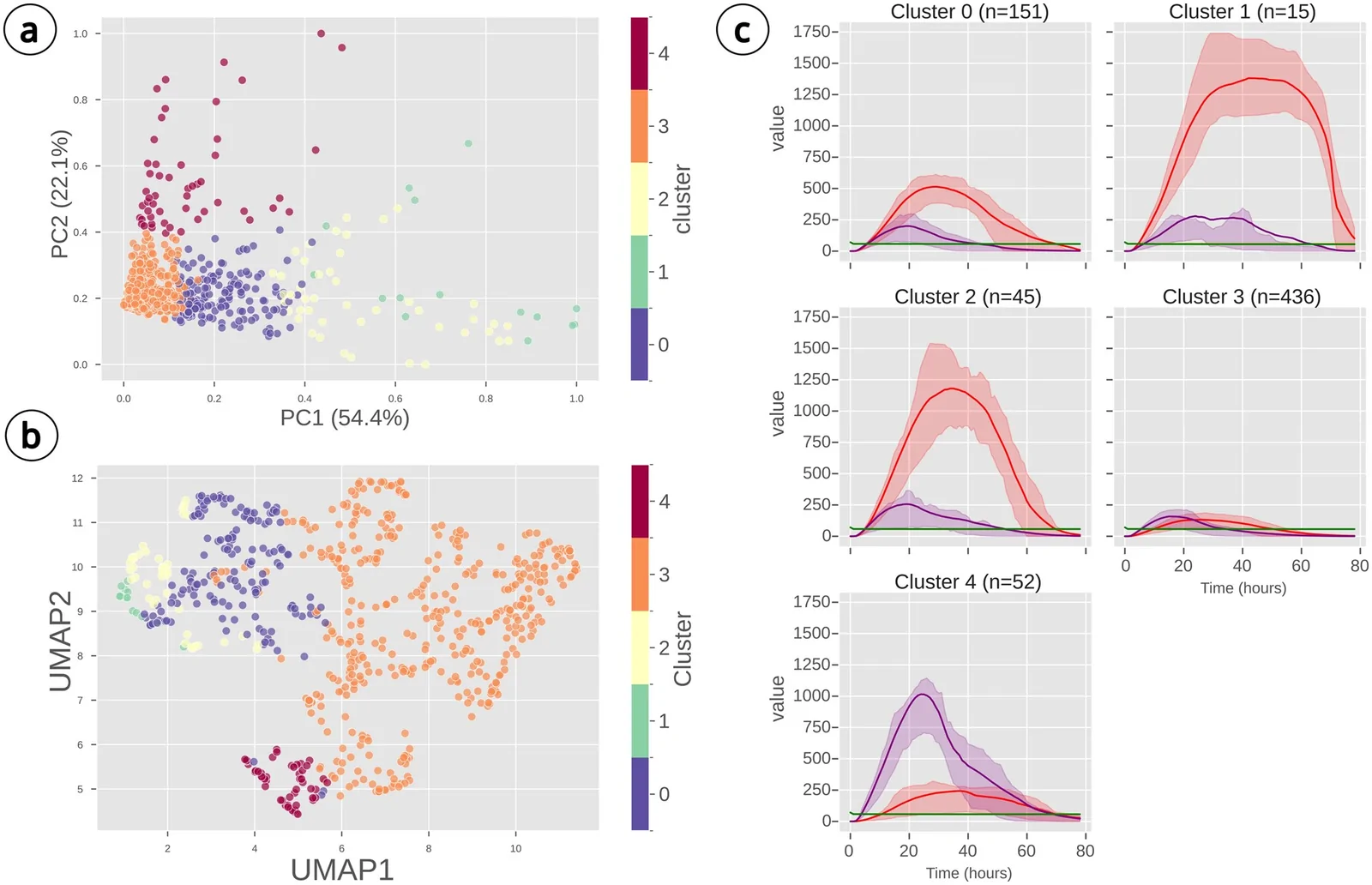
Clusters identification among the best simulations. (a) PCA from the best simulation (score = 10/10) kinetics. Clusters were identified with k-means method with n_cluster parameter determined using the bootstrap elbow method (*n* = 100). (b) 2D UMAP drawn from the best simulations’ *n* dimensions PCA. (c) Mean curves and standard deviations of agent populations for each clusters.

PCA was applied to simulation kinetics satisfying all defined criteria. The reduced space did not reveal sharply separated clusters, indicating that plausible trajectories lie on a continuum (Fig. 4a). However, k-means clustering identified reasonably stable groups of kinetic profiles with distinct temporal organizations (Fig. 4b, Fig. S3, available as supplementary data at *Bioinformatics* online). Clusters differed in both amplitude and timing of the inflammatory response (Fig. 4c). In certain regimes, macrophage-driven processes structured resolution, while in others neutrophil kinetics played a dominant role. These differences reflect not simple scaling effects but qualitatively distinct coordination patterns between recruitment, polarization, and clearance. Thus, even within the constrained subspace compatible with canonical inflammation, multiple coherent dynamical organizations coexist.

From a systems perspective, sterile inflammation therefore corresponds not to a single optimal trajectory but to a family of functionally equivalent regimes balancing pro-inflammatory amplification and pro-resolving feedback while restoring tissue integrity. Such multiplicity may underlie inter-individual and tissue-specific variability and suggests that subtle shifts between regimes could contribute to chronic low-grade inflammation without complete regulatory failure.

## 4 Conclusion/discussion

We developed an agent-based model of inflammation using a mono-agent framework that captures functional redundancy in immune processes along a continuous pro- to anti-inflammatory axis. By abstracting immune cell functions, the model allows exploration of compensatory and cooperative behaviors in a spatial tissue context.

Global sensitivity analysis showed high potential, producing diverse inflammatory trajectories. Expert-driven filtering confirmed that this space includes dynamics consistent with canonical sterile inflammation profiles, including proper recruitment timing, signal transitions, and resolution patterns, supporting the model’s internal coherence and its ability to represent varied inflammatory regimes.

The mono-agent paradigm offers a useful framework for contexts with reduced immune populations, where immune cells share the same functional modules but differ by their initial parameters, which define cells behaviors. However, this abstraction may induce artificial compensations between immune subtypes as LHS was performed without constraints between cell type parameters. This, combined with the broad parameter ranges selected in accordance with the early stage of model analysis, could explain the low validation rate observed among the parameter combinations tested using LHS. Thus, parameter dimension reduction, as well as refinement of the evaluation criteria, is needed. The stochastic nature of ABMs also calls for the implementation of an uncertainty analysis to better characterize the specific effects of parameter configurations.

Much information from simulations remains untapped. Current clustering uses only population-level kinetics, whereas individual polarization, spatial organization, tissue residence, and phagocytic activity per agent could refine trajectory classification and improve interpretability. In addition, a better characterization of MSC-specific behavior will be required for future studies. In tissues with a low abundance of resident macrophages and high exposure to inflammatory conditions [e.g. periodontal tissues (Almubarak *et al.* 2020)], MSCs may compensate for the reduced immune cell presence (Planat-Benard *et al.* 2021). The model provides a framework to investigate whether MSCs primarily modulate the immune response through their known immunoregulatory properties, or whether they adopt macrophage-like functional behaviors within the inflammatory process.

Future work should also focus on more tightly linking the agent-based model to biological data by refining evaluation criteria based on experimentally measurable outputs. As cytometry datasets often lack absolute cell counts, validation may need to rely on temporal ratios, while scRNA-seq data could provide additional constraints on the parameter space. Additional perturbation scenarios are also needed to assess robustness and tissue heterogeneity, particularly varying initial proportions of non-generic cell types to capture diverse inflammatory niches. LHS combined with filtering rules allows the identification of plausible inflammatory scenarios without relying on experimental data. To further demonstrate the biological relevance of these scenarios, the model can subsequently be calibrated using different experimental datasets (e.g. cell types or cytokines kinetics, polarization indices…). This approach would enable a more precise characterization of the plausible scenario space and could ultimately help illustrate the trajectories leading to impaired resolution, such as those observed during inflammaging.

Overall, this work establishes a flexible, spatially explicit framework for sterile inflammation and enables data-informed exploration of large dynamical solution spaces from a systems biology perspective.

## Supplementary Material

btag533_Supplementary_Data

## Data Availability

The data that support the findings of this study are available on this page: GitLab and from the corresponding author upon reasonable request.
