# Supplementary material for: Agent-based modeling identifies multiple paths for inflammation resolution
Source: Bioinformatics. 2026 Aug 19;42(Suppl 2):btag533. doi: 10.1093/bioinformatics/btag533 (PMC13501331; doi:10.1093/bioinformatics/btag533)
Supplement: Supplementary file 1 [file btag533_supplementary_data.pdf]

Supplementary Material

Supplementary data for:

Agent Based Modelling Identify many paths for Inflammation Resolution

Hermès Desgrez–Dautet, David Bernard, Béatrice Cousin, Paul Monsarrat, Sylvain Cussat-Blanc

Journal Name (Year)

1. Supplementary Data

1.1. Supplementary Tables

Cell type	function	src
Circulating macrophage	polarization pro-infla pro-reso	[1, 2]
	produce inflammatory signal	[3, 1]
	produce resolutive signal	[3, 1]
	phagocytosis/efferocytosis	[4, 1]
	chemotaxis	[1]
Resident macrophage	polarization pro-infla pro-reso	[1, 2]
	produce inflammatory signal	[1]
	produce resolutive signal	[1]
	phagocytosis/efferocytosis	[4, 1]
	chemotaxis	[1]
Neutrophile	polarization pro-infla pro-reso	[5, 6]
	produce inflammatory signal	[7]
	produce resolutive signal	[7, 5, 6]
	phagocytosis/efferocytosis	[8]
	chemotaxis	[9, 10]
MSCs	polarization pro-infla pro-reso	[11, 12]
	produce inflammatory signal	[11]
	produce resolutive signal	[11]
	phagocytosis/efferocytosis	[13]
	chemotaxis	[14]

Table S1: **Activated cell associated redondant functions**

	Circulating Mph	Resident Mph	Neutrophils	MSC	Other	Vessel
cellOccupation	0.15	0.15	0.05	0.1	0.1	0.2
dangerImpactOnPol	0.1	0.1	0.2	0.1	-	-
deltaPolInfla	0.05	0.05	0.2	0.05	-	-
deltaPolReso	0.1	0.2	0.05	0.25	-	-
inactivityPolTh	0.09	0.09	0.05	0.09	-	-
infResBalanceImpactOnApopPb	0.0005	0.0005	0.01	0.005	0.005	0.01
infResBalanceImpactOnNecroPb	0.1	0.1	0.00001	0.00001	0.0001	0.0001
lifeImpact	0.2	0.2	0.01	0.1	-	-
maxPolarisation	1.5	1.5	1.5	1.5	-	-
phagoImpactOnPol	0.5	0.5	0.2	0.5	-	-
pitoidGap	0.3	0.3	0.3	0.3	-	-
pitoidSlope	8	8	8	8	-	-
polarBias	0.01	-0.01	0.05	-0.02	-	-
polarDecayInfla	0.1	0.1	0.05	0.2	-	-
polarDecayReso	0.01	0.01	0.01	0.01	-	-
probaMoveSpeed	0.5	0.2	0.9	0.2	-	-
signalApopProd	0.2	0.2	0.2	0.2	0.2	0.2
signalDangerProd	0.3	0.3	0.3	0.3	0.3	0.3
signalInflaProd	0.1	0.1	0.2	0.05	-	-
signalInflaProdNecrosis	0.1	0.1	0.1	0.025	0.01	0.01
signalResoProd	0.15	0.2	0.01	0.3	-	-
thAPOP	0.01	0.01	0.01	0.01	0.01	0.01
thDANGER	0.01	0.01	0.01	0.01	0.01	0.01
thINFLA	0.1	0.1	0.1	0.1	0.1	0.3
timeUntilDecay	5	5	5	5	-	-
pbSpeedRecruitmentCirculant	-	-	-	-	-	1
pbSpeedRecruitmentNeutro	-	-	-	-	-	5

Table S2: **Scenario testing cell agents parameters.**

Scenario parameters	value
diff_apopCoef	0.7
diff_apopEvap	0.05
diff_dangerCoef	0.7
diff_dangerEvap	0.05
diff_inflaCoef	0.7
diff_inflaEvap	0.05
diff_resoCoef	0.7
diff_resoEvap	0.05
gridSize	40
rateMacro	0.04
rateStromal	0.04
rateVessel	0.02
dt	360
maxStep	800

Table S3: **Scenario parameters LHS ranges**

Parameters	Circ. Mph		Res. Mph		Neutrophils		MSC		Other		Vessel	
	min	max	min	max	min	max	min	max	min	max	min	max
cellOccupation	0.05	0.5	0.05	0.5	0.05	0.5	0.05	0.5	0.05	0.5	0.05	0.5
dangerImpactOnPol	0	2	0	2	0	2	0	2	-	-	-	-
deltaPolInfla	0.001	0.5	0.001	0.5	0.001	0.5	0.001	0.5	-	-	-	-
deltaPolReso	0.001	0.5	0.001	0.5	0.001	0.5	0.001	0.5	-	-	-	-
inactivityPolTh	0.01	0.5	0.01	0.5	0.01	0.5	0.01	0.5	-	-	-	-
infResBalImpactOnApopPb[1]	0	0.1	0	0.1	0	0.1	0	0.1	0	0.1	0	0.1
infResBalImpactOnNecroPb[1]	0	0.1	0	0.1	0	0.1	0	0.1	0	0.1	0	0.1
lifeImpact	0	1	0	1	0	1	0	1	-	-	-	-
phagoImpactOnPol	0	2	0	2	0	2	0	2	-	-	-	-
pitoidGap	0	1	0	1	0	1	0	1	-	-	-	-
pitoidSlope[2]	1	15	1	15	1	15	1	15	-	-	-	-
polarBias	-0.5	0.5	-0.5	0.5	-0.5	0.5	-0.5	0.5	-	-	-	-
polarDecayInfla	0.01	0.5	0.01	0.5	0.01	0.5	0.01	0.5	-	-	-	-
polarDecayReso	0.01	0.5	0.01	0.5	0.01	0.5	0.01	0.5	-	-	-	-
probaMoveSpeed	0.1	1	0.1	1	0.1	1	0.1	1	0	0	-	-
signalApopProd	0	2	0	2	0	2	0	2	0	2	0	2
signalDangerProd	0	2	0	2	0	2	0	2	0	2	0	2
signalInflaProd	0	2	0	2	0	2	0	2	-	-	-	-
signalInflaProdNecrosis	0	2	0	2	0	2	0	2	0	2	0	2
signalResoProd	0	2	0	2	0	2	0	2	-	-	-	-
thAPOP	0	1	0	1	0	1	0	1	0	1	0	1
thDANGER	0	1	0	1	0	1	0	1	0	1	0	1
thINFLA	0	1	0	1	0	1	0	1	0	1	0	1
timeUntilDecay[2]	1	10	1	10	1	10	1	10	-	-	-	-
pbSpeedRecruitCirculant	-	-	-	-	-	-	-	-	-	-	0	5
pbSpeedRecruitNeutro	-	-	-	-	-	-	-	-	-	-	0	5

Table S4: **Parameters LHS ranges.** [1] Logarithmic exploration. [2] Integer exploration.

Scenario parameters	min	max
diff__apopCoef	0.5	0.9
diff__apopEvap	0.01	0.1
diff__dangerCoef	0.5	0.9
diff__dangerEvap	0.01	0.1
diff__inflaCoef	0.5	0.9
diff__inflaEvap	0.01	0.1
diff__resoCoef	0.5	0.09
diff__resoEvap	0.01	0.1
gridSize	40*	
rateMacro	0.04*	
rateStromal	0.04*	
rateVessel	0.02*	
dt	360*	
maxStep	800*	

Table S5: **Scenario parameters LHS ranges** * fixed parameters

1.2. Supplementary Figures

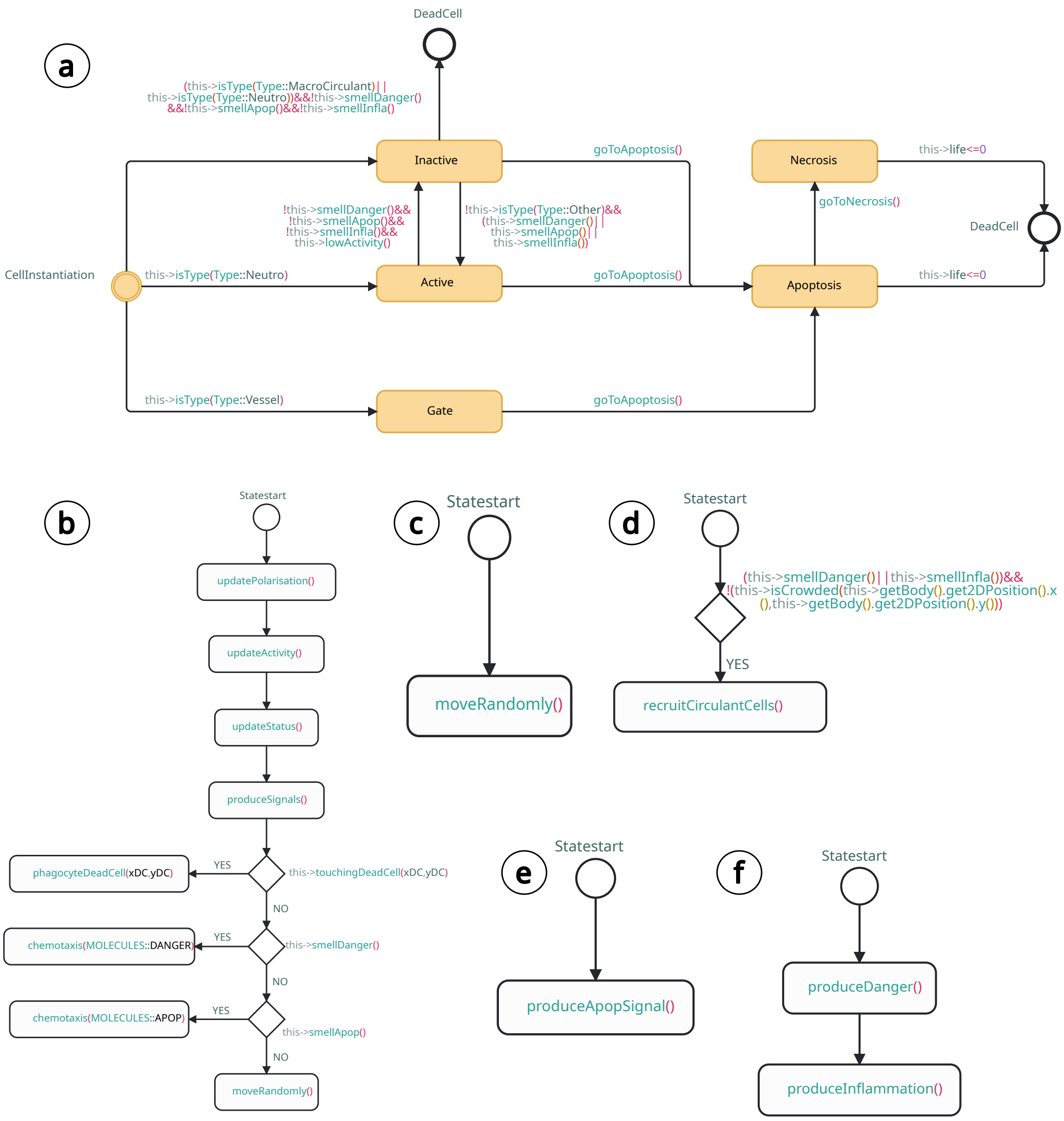

Figure S1: **Model graphical representation.** (a) State Transition diagram of the whole model. (b) Activity diagram of Active state. (c) Activity diagram of Inactive state. (d) Activity diagram of Gate state. (e) Activity diagram of Apoptosis state. (f) Activity diagram of Necrosis state.

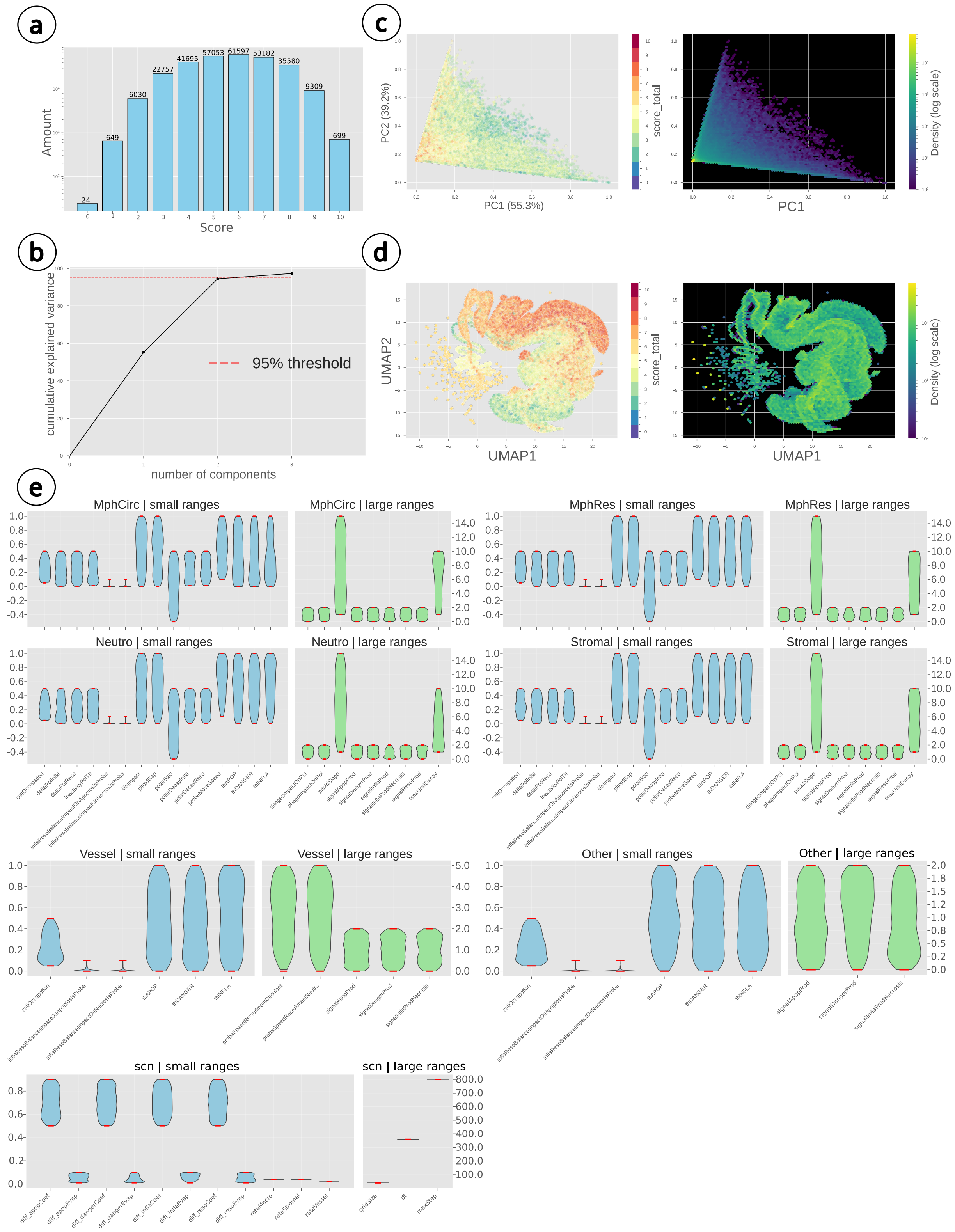

Figure S2: **UMAP generation.** PCA was generated from immune cells agents kinetics vector. (a) Histogram of score distribution across the dataset. (b) Cumulated variance explanation per component, red dotted line is 95% threshold. (c) PCA scatterplot (PC1 and PC2), colorized by total score (left), colorized by local density (right). (d) UMAP scatterplot, 2D UMAP is generated on PCA based on PCA n components, colorized by total score (left), colorized by local density (right). (e) Distributions of parameters that associated with best total score (10/10), blue violin plots refers to left scale, green violin refer to right scale, red bars are min and max of LHS outputs (full parameter set).

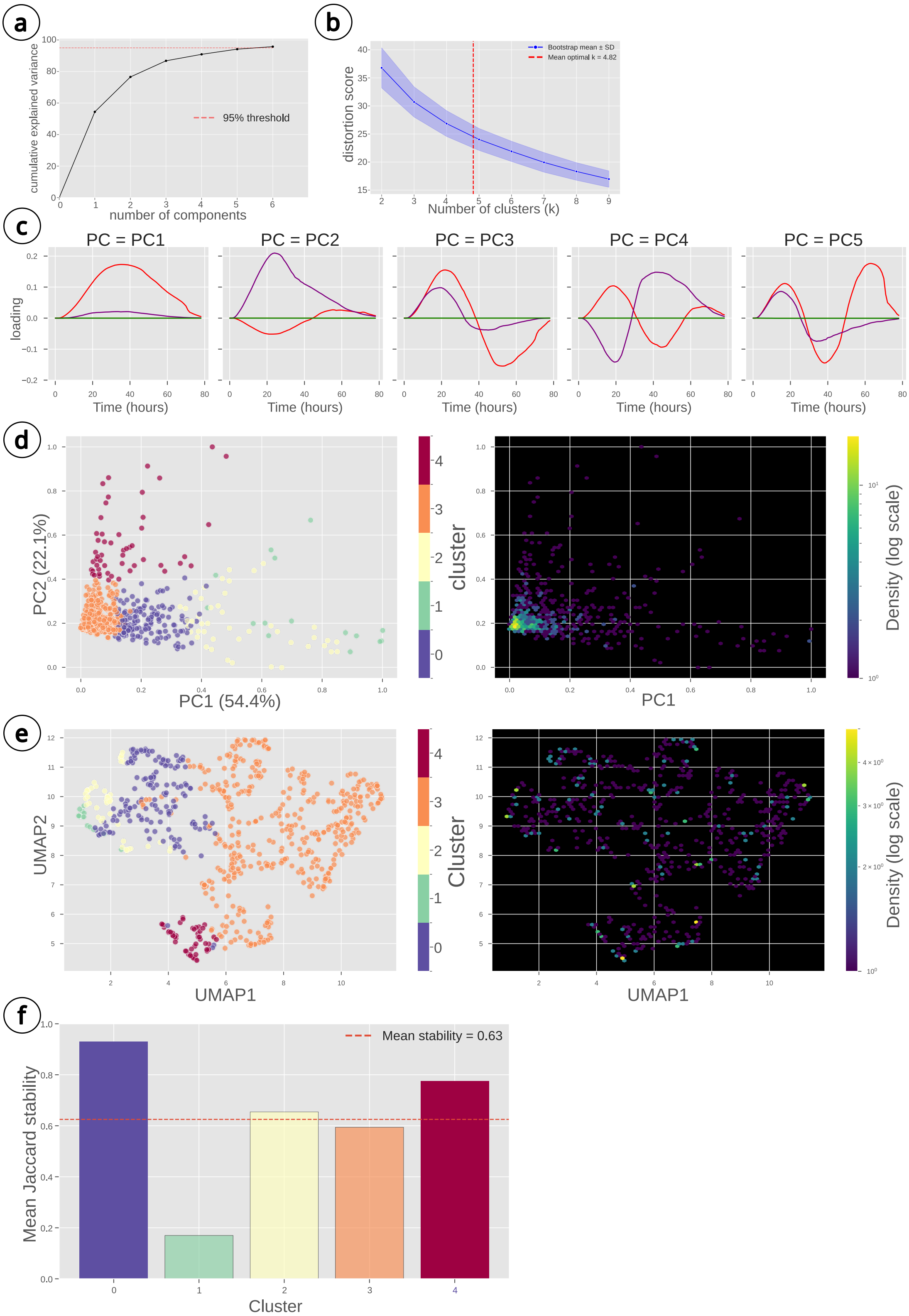

Figure S3: **Clustering data.** Clustering was performed on PCA reduced data. (a) Cumulated variance explanation per component, red dotted line is 95% threshold. (b) Elbow scores in function of the number of cluster (k with KElbowVisualizer method), vertical line locate the mean k_score (n=100). (c) Principal components values for resident (orange) and circulating (red) macrophages, neutrophils (purple) and stromal cells (green). (d) PCA scatterplot (PC1 and PC2), colorized by cluster id (left), colorized by local density (right). (e) UMAP scatterplot, 2D UMAP is generated on PCA based on PCA n components, colorized by cluster id (left), colorized by local density (right). (f) Jaccard scores calculated for every clusters.

2. Supplementary methods

Because the model was designed to remain flexible and cover a broad range of inflammatory scenarios, it cannot initially rely on specific experimental data constraints. Nevertheless, defining criteria to distinguish plausible from implausible simulations remains necessary. We therefore established a rule-based scoring framework to evaluate simulation outcomes.

2.1. Rule 1 : Neutrophils max timing

Neutrophils peak has to fit in a certain timing (i.e. 6 to 48h) to replicate classical inflammation. Thus, we choose to test whether maximum neutrophil count occurs in this range.

Let $N_{t\,\max}$ the timing at which maximum number of neutrophils is reach. A simulation passes the neutrophil timing criterion if

$$6h \leq N_{tmax} \leq 48$$

2.2. Rule 2 : Circulating cells recruitment order

Neutrophils and circulating macrophages (or monocyte) are recruited in a well described sequence. Thus, we ensured that neutrophils enter the tissue prior to circulating macrophages by extracting weighted mean values of neutrophils and circulating macrophages time series.

Let $\bar{N_t}$ and $\bar{M}_{c,t}$ denote the timing of neutrophils and circulating macrophages weighted average (weight = cell amount, with numpy 2.4 average function and weights option).

We enforced the biologically expected recruitment order by requiring

$$\bar{N_t} < \bar{M}_{c,t}$$

2.3. Rule 3 : Amount of neutrophils during simulation

Neutrophils recruitment is constitutive of inflammation process. Thus, to ensure a significant neutrophil presence throughout the simulation, the maximum number of neutrophils was compared to the maximum number of resident macrophages (stable across all simulations).

Let $N_{\max}$, $M_{r,\max}$ the neutrophils and resident macrophages maximums (post lesion).

To ensure biologically plausible neutrophils quantity, we imposed

$$M_{r,\max} \leq N_{\max} \leq 50M_{r,\max}$$

The 50 factor is expected to maintain neutrophils to a close order of magnitude of resident macrophages while assuring sufficient recruitment.

2.4. Rule 4 : Amount of circulating macrophages during simulation

Similarely to neutrophils, circulating macrophages (including monocytes) have to be recruited for the inflammatory process. Thus, to ensure a significant circulating macrophage presence throughout the simulation, the maximum number of those was compared to the maximum number of resident macrophages (stable across all simulations).

Let $M_{c,\max}$, $M_{r,\max}$ the circulating and resident macrophages maximums (post lesion).

To ensure biologically plausible quantity, we imposed

$$M_{r,\max} \leq M_{c,\max} \leq 50 M_{r,\max}$$

The 50 factor is expected to maintain circulating macrophages to a close order of magnitude of resident macrophages while assuring sufficient recruitment.

2.5. Rule 5 : Amount of inflammatory cells

at the end

To verify that inflammation resolves, we compared the number of polarized inflammatory cells at the end of the simulation to their maximum observed number.

Let $P_{infla,\max}$ and $P_{infla,end}$ the maximum and the last number of inflammatory polarized cells. To validate the resolution, we imposed

$$P_{infla,end} \leq 0.1 P_{infla,\max}$$

The 0.1 threshold forces the number of inflammatory cell to drop before the end of the simulation without cutting too hard if only few cells remain.

2.6. Rule 6 : Neutrophils amount at the end

To assess neutrophil clearance via apoptosis or tissue egress, we compared the maximum neutrophil count to the number remaining at the end of the simulation.

Let $N_{\max}$ and N_{end} denote the maximum and the last number of neutrophils during simulation. The simulation passes the neutrophil clearance criterion if

$$N_{end} \leq 0.1 N_{\max}$$

The 0.1 threshold forces the number of neutrophil quantity to drop before the end of the simulation without cutting too hard if only few cells remain.

2.7. Rule 7 : Dead cell amount at the end

To verify clearance of the initial danger source, we compared the maximum number of necrotic cells to the number remaining at the end of the simulation.

Let $D_{\max}$ and D_{end} denote, respectively, the maximum number of necrotic cells and the number at the end of the simulation, The simulation passes the necrotic cell clearance criterion if

$$D_{end} \leq 0.1 D_{\max}.$$

The 0.1 threshold forces the number of inflammatory cell to drop before the end of the simulation without cutting too hard if only few cells remain.

2.8. Rule 8 : Resident immune cell amount at the end

To ensure preservation of resident cells, we verified that the initial stocks (post lesion, step=10) of resident macrophages and MSCs do not decrease drastically by the end of the simulation (step=799).

Let $M_{r,\max}$ and $M_{r,end}$ respectively the number of resident macrophage post lesion and at the end of the simulation, and $S_{\max}$ and S_{end} respectively the number of MSCs post lesion and at the end of the simulation. Then the initial resident cell number and the loosed quantity are noted

$$Res_{\max} = M_{r,\max} + S_{\max}, \quad Res_{lost} = Res_{\max} - (M_{r,end} + S_{end}),$$

The simulation passes the resident cells preservation criterion if

$$Res_{lost} \leq 0.1 Res_{\max}$$

The 0.1 threshold ensures that some resident cells remain by the end of the simulation.

2.9. Rule 9 : Tissue loss at the end

To ensure preservation of local tissue integrity, we verified that all local cell populations do not decrease drastically by the end of the simulation, excluding dead cells. Let $M_{r,\max}$, $M_{r,end}$, $S_{\max}$, S_{end}, $O_{\max}$, O_{end}, and $V_{\max}$, V_{end} denote the numbers of resident macrophages, stromal cells, other local cells, and vessel cells, respectively, after lesion induction and at the end of the simulation. Define the initial and final counts as

$$R_{\max} = M_{r,\max} + S_{\max} + O_{\max} + V_{\max} \ , \quad R_{end} = M_{r,end} + S_{end} + O_{end} + V_{end}$$

The simulation passes the local cell preservation criterion if

$$R_{end} \geq 0.5 R_{\max}$$

The 0.5 threshold suppress the tissue that have been too much harmed by the inflammation.

2.10. Rule 10 : inflammatory and resolutive signals sequence

To ensure consistency of signaling dynamics, we verified that the inflammatory signal precedes the resolution signal. Let $\bar{Sig}_{infla,t}$ and $\bar{Sig}_{reso,t}$ denote the inflammatory and resolution signals weighted average (similarly as Rule 2).

We enforced the biologically expected recruitment order by requiring

$$\bar{Sig}_{infla,t} < \bar{Sig}_{reso,t}$$

3. Supplementary Data Availability

The data and scripts used to produce supplementary data are available from the corresponding author on GitLab page and upon reasonable request

References

[1] E. A. Ross, A. Devitt, and J. R. Johnson, “Macrophages: The Good, the Bad, and the Gluttony,” *Frontiers in Immunology*, vol. 12, 2021.

[2] Z. Strizova, I. Benesova, R. Bartolini, R. Novysedlak, E. Cecrdlova, L. K. Foley, and I. Striz, "M1/M2 macrophages and their overlaps – myth or reality?" *Clinical Science (London, England : 1979)*, vol. 137, no. 15, pp. 1067–1093, 2023.

[3] P. Italiani, E. M. C. Mazza, D. Lucchesi, I. Cifola, C. Gemelli, A. Grande, C. Battaglia, S. Bicciato, and D. Boraschi, "Transcriptomic Profiling of the Development of the Inflammatory Response in Human Monocytes In Vitro," *PLOS ONE*, vol. 9, no. 2, p. e87680, 2014.

[4] A. L. Lam and B. Heit, "Having an Old Friend for Dinner: The Interplay between Apoptotic Cells and Efferocytes," *Cells*, vol. 10, no. 5, p. 1265, 2021.

[5] W. Loh and S. Vermeren, "Anti-Inflammatory Neutrophil Functions in the Resolution of Inflammation and Tissue Repair," *Cells*, vol. 11, no. 24, p. 4076, 2022.

[6] A. C. Mihaila, L. Ciortan, M. M. Tucureanu, M. Simionescu, and E. Butoi, "Anti-Inflammatory Neutrophils Reprogram Macrophages toward a Pro-Healing Phenotype with Increased Efferocytosis Capacity," *Cells*, vol. 13, no. 3, p. 208, 2024.

[7] S. Shrestha and C.-W. Hong, "Extracellular Mechanisms of Neutrophils in Immune Cell Crosstalk," *Immune Network*, vol. 23, no. 5, p. e38, 2023.

[8] R. Herro and H. L. Grimes, "The diverse roles of neutrophils from protection to pathogenesis," *Nature Immunology*, vol. 25, no. 12, pp. 2209–2219, 2024.

[9] B. McDonald, K. Pittman, G. B. Menezes, S. A. Hirota, I. Slaba, C. C. M. Waterhouse, P. L. Beck, D. A. Muruve, and P. Kubes, "Intravascular Danger Signals Guide Neutrophils to Sites of Sterile Inflammation," *Science*, vol. 330, no. 6002, pp. 362–366, 2010.

[10] de Oliveira Sofia, R. E. E., and H. Anna, "Neutrophil migration in infection and wound repair: Going forward in reverse," *Nature reviews. Immunology*, vol. 16, no. 6, pp. 378–391, 2016.

[11] R. S. Waterman, S. L. Tomchuck, S. L. Henkle, and A. M. Betancourt, "A New Mesenchymal Stem Cell (MSC) Paradigm: Polarization into a Pro-Inflammatory MSC1 or an Immunosuppressive MSC2 Phenotype," *PLOS ONE*, vol. 5, no. 4, p. e10088, 2010.

[12] V. Planat-Benard, A. Varin, and L. Casteilla, "MSCs and Inflammatory Cells Crosstalk in Regenerative Medicine: Concerted Actions for Optimized Resolution Driven by Energy Metabolism," *Frontiers in Immunology*, vol. 12, p. 626755, 2021.

[13] B. Cousin, M. André, L. Casteilla, and L. Pénicaud, "Altered macrophage-like functions of preadipocytes in inflammation and genetic obesity," *Journal of Cellular Physiology*, vol. 186, no. 3, pp. 380–386, 2001.

[14] Z. Xie, W. Yu, G. Zheng, J. Li, S. Cen, G. Ye, Z. Li, W. Liu, M. Li, J. Lin, Z. Su, Y. Che, F. Ye, P. Wang, Y. Wu, and H. Shen, "TNF-α-mediated m6A modification of ELMO1 triggers directional migration of mesenchymal stem cell in ankylosing spondylitis," *Nature Communications*, vol. 12, p. 5373, 2021.
